# Acetozone in the Therapy of Typhoid Fever

**Published:** 1902-11

**Authors:** 


### Acetozone in the Therapy of Typhoid Fever.

The recent discovery, by Duval and Bassett, of the presence of the bacillus dysenteriæ (Shiga) in forty cases of infantile summer diarrhea, awakens renewed interest in the subject of intestinal antisepsis. But a few months have elapsed since Drs. P. C. Freer and F. G. Novy, of the University of Michigan, demonstrated the enormous germicidal power of benzoyl-acetyl-peroxide, more familiarly known as acezotone Although the preliminary reports of these investigators were of necessity based upon results of laboratory experiments, their expectations are already being realised in clinical work, particularly in the treatment of typhoid fever.

In Chicago, where a large number of cases of typhoid have been reported, acezotone has been used exclusively in the treatment of about 300 of them. The consensus of opinion is that it causes the temperature to decline earlier than usual in the course of the disease, and it ameliorates the mental and physical condition of the patient, in all probability by controlling the toxemia.

Two Chicago physicians, I. A. Abt and E. Lackner, have thus far reported (*Therapeutic Gazette*, October, 1901,) 40 cases of typhoid in children treated with acetozone, with but two deaths, a mortality of 5 per cent. One of the patients that died succumbed to pneumonia and pulmonary edema, the other to great pyrexia on the fifth day. Stupor and tympanites were almost entirely absent in all the cases; the characteristic typhoid fetor of the stools was markedly diminished, and the hemorrhage occurred but twice, and in the same case. The average duration of the febrile period, in 37 cases, after beginning acetozone treatment, was 13½ days. The drug did not seem to act upon the heart or respiratory apparatus.

Early this year Eugene Wasdin, M. D., of the U. S. Marine Hospital Service, Buffalo, N. Y., reported 27 cases (*American Medicine*, February 8, 1902,) of typhoid fever, 24 of which were treated with acezotone, all of the patients recovering. The writer says:

Its application in typhoid fever has been followed by very happy results; its use has been directed to the destruction of the germ in its primary lung colony and also in its secondary intestinal colony, and it has been used by hypodermoclysis to combat terminal expressions, with the result that in 24 cases the disease has been limited almost entirely to the expression of intoxication from the primary focus, the intestinal symptoms remaining entirely in abeyance, and the disease has been shorn of many of its most disagreeable features.

In a second paper, which appeared in the *Therapeutic Gazette*, May 15, 1902, the same writer states that his patients were given from 1,500 to 2,000 cc. of the aqueous solution of acetozone daily. The diet was milk diluted with the same solution. The first influence of the drug is observed in the increased secretion of urine. That this is not due wholly to the ingestion of large quantities of water necessitated by the use of the saturated solution is evident from the author's assertion that the same result was observed when acetozone was administered in capsules. The second influence to which attention is directed is the very pronounced decrease of the odor of the stools, while plate cultures from the dejecta showed comparatively few germs.

The deodorant and diuretic effects of acetozone were also observed by G. H. Westinghouse, M. D., of Buffalo (BUFFALO MEDICAL JOURNAL, August, 1902, *q. v.*,) who used it in seven cases.

---

PERCUSSION OF THE LOWER BORDER OF THE LIVER.—Abrams (*Medical News*, February 8, 1902,) asserts that it is never safe to declare the liver enlarged unless it is palpable. He recommends the following simple procedure for mapping out the lower border of the liver: the patient should incline his body backward as far as possible, while supporting himself, with his hands resting on his hips. Then percussion, which must be light, will determine the lower border of the liver with little difficulty. Strong percussion will cause neighboring hollow organs to vibrate. The finger must be firmly imbedded in the abdominal wall, otherwise the blow will not be transmitted to the liver. The bowels must be thoroughly evacuated before the examination.

